# Limbal Stem Cells from Aged Donors Are a Suitable Source for Clinical Application

**DOI:** 10.1155/2016/3032128

**Published:** 2016-11-30

**Authors:** Nuria Nieto-Nicolau, Eva M. Martínez-Conesa, Ricardo P. Casaroli-Marano

**Affiliations:** ^1^CellTec-UB, Department of Cell Biology, University of Barcelona, Barcelona, Spain; ^2^Barcelona Tissue Bank (BTB), Banc de Sang I Teixits (BST, GenCAT), Barcelona, Spain; ^3^Department of Surgery, School of Medicine & Hospital Clinic of Barcelona (IDIBAPS), University of Barcelona, Barcelona, Spain

## Abstract

Limbal stem cells (LSC) are the progenitor cells that maintain the transparency of the cornea. Limbal stem cell deficiency (LSCD) leads to corneal opacity, inflammation, scarring, and blindness. A clinical approach to treat this condition consists in LSC transplantation (LSCT) after* ex vivo* expansion of LSC. In unilateral LSCD, an autologous transplant is possible, but cases of bilateral LSCD require allogenic LSCT. Cadaveric donors represent the most important source of LSC allografts for treatment of bilateral LSCD when living relative donors are not available. To evaluate the suitability of aged cadaveric donors for LSCT, we compared three pools of LSC from donors of different ages (<60 years, 60–75 years, and >75 years). We evaluated graft quality in terms of percent of p63-positive (p63+) cells by immunofluorescence, colony forming efficiency, and mRNA and protein expression of p63, PAX6, Wnt7a, E-cadherin, and cytokeratin (CK) 12, CK3, and CK19. The results showed that LSC cultures from aged donors can express ≥3% of p63+ cells—considered as the minimum value for predicting favorable clinical outcomes after LSCT—suggesting that these cells could be a suitable source of LSC for transplantation. Our results also indicate the need to evaluate LSC graft quality criteria for each donor.

## 1. Introduction

The cornea is a transparent, avascular, stratified tissue covered by a highly specialized epithelium, the integrity of which depends on a group of stem cells in the basal epithelial layer of the limbal region [1]. These cells are called limbal stem cells (LSC), and their depletion causes corneal blindness. This state of limbal stem cell deficiency (LSCD) is associated with a loss of corneal transparency because of conjunctivalization and new-vessel formation on the cornea [2]. LSCD affects approximately 10 million people worldwide [3, 4]. Initially, one clinical approach was the limbal transplantation (autologous or allogenic), in which a large biopsy of the limbic region of a healthy eye was transplanted to the damaged eye. This technique induced the risk of LSCD on the healthy eye in cases of autologous tissue [5, 6]. Overcoming this risk, cultured limbal epithelial stem cell transplantation (CLET) has become a more common and less invasive clinical approach [6]. With CLET, LSC from a minimally invasive limbal biopsy are seeded on a biocompatible carrier for* ex vivo* culture expansion and transplantation [7]. Keratoplasty may then be performed to recover the vision of the damaged eye [8]. In patients with unilateral LSCD, the LSC for CLET can usually be sourced from the patient's healthy eye; however, cases of bilateral LSCD are far more challenging because autologous LSC are not available. Thus, it is important both to seek new sources of stem cells with the potential to transdifferentiate to corneal epithelium [9] and to improve current allogenic transplantation techniques to resolve these issues with bilateral LSCD. Moreover, CLET grafts could also be obtained for allogenic transplantation from a living relative or from a cadaveric donor when compatible relatives are not available [10]. However, with these approaches, systemic immunosuppression is recommended to avoid tissue rejection [6]. This, along with a careful selection of patients, allows allogenic CLET to achieve an equal success rate compared to autotransplantation [7, 8, 11].

Several therapeutic approaches with alternative stem cells, such as mesenchymal stem cells (MSC) [12–16], cultured oral mucosa epithelial cells [17, 18], embryonic stem cells (ESC) [19], or induced pluripotent stem cells (IPSC) [20–22], have been established to either study their potential to differentiate into corneal epithelium phenotypes or to reconstruct a damaged corneal epithelium in experimental models. However, several key issues remain unresolved and these options require a great deal of development before they are ready for clinical application in humans. For example, the potential of MSC to differentiate fully to corneal epithelium is unclear; although MSC express low levels of corneal cytokeratins, these levels are always lower than for corneal epithelium [23]. Equally important is the fact that long-term outcomes from cultured oral mucosa epithelial cell transplantation have not yet been assessed [17, 24]. Also, the use of ESC has important ethical implications, whereas factors associated with IPSC generation have been linked to oncogenic transformation [25]. While further investigation is needed with these alternative sources of stem cells, we should strive to increase the efficiency of transplantation with allogenic LSC to improve the treatment of bilateral LSCD. Enhancing the efficiency of transplantation is a major research concern, with many attempts having been made to identify the optimal LSC culture techniques and surgical approaches [11].

Given the need of donors for allotransplantation and in order to expand the donor pool, we propose to explore the possibility that normal corneas from aged cadaveric donors could represent a suitable source of LSC for clinical application. To do so, we characterized pools of LSC from donors in three different age ranges: <60 years, 60–75 years, and >75 years. We evaluated these cells using the following well-established criteria for assessing graft quality: quantification of p63-positive (p63+) cells because the presence of ≥3% p63+ cells has been associated with better LSCT outcomes [26]; corneal and conjunctival cell markers [27–31]; and the colony forming efficiency (CFE).

## 2. Materials and Methods

### 2.1. Cell Cultures

Murine 3T3 Swiss albino fibroblasts (3T3-SA, CCL-92) were obtained from the American Type Culture Collection (ATCC, Manassas, VA). Before use, cells were inactivated by irradiation with 6000 rads. After this, cells were plated onto culture dishes at 2 × 10^4^ cells/cm^2^ for feeder-layer use.

### 2.2. Human LSC, Corneal Epithelial Cells, and Conjunctival Epithelial Cells

Cadaveric adult human limbal tissue from different donors was obtained from the Eye Bank of Centro de Oftalmología Barraquer (Barcelona, Spain; http://www.barraquer.com/en/) and Barcelona Tissue Bank (BTB-BST, Barcelona, Spain; http://www.bancsang.net/en_index/). The informed consent for the use of tissues for experimental purposes was obtained in accordance with the requirements of the Declaration of Helsinki and local laws. Any active transmissible infections were excluded by serologic analyses. All tissues were used 18–24 hrs after donor death. LSC were isolated as previously described [32, 33] and cultured until subconfluence with supplemented hormonal epithelial media (SHEM) consisting of the following: Dulbecco's modified Eagle's medium/Ham's and F-12 (2 : 1 vol : vol) mixture (DMEM/F12; Invitrogen, Carlsbad, CA) supplemented with 2 mM L-glutamine (Lonza, Verviers, Belgium), 5 *μ*g/mL insulin (Sigma-Aldrich, Munich, Germany), 10 ng/mL epidermal growth factor (hEGF, Sigma-Aldrich), 0.5% dimethyl sulfoxide (DMSO, Sigma-Aldrich), 0.4 *μ*g/mL hydrocortisone (Sigma-Aldrich), 2 nM triiodothyronine (Sigma-Aldrich), and 0.18 mM adenine (Sigma-Aldrich), with 10% FCS and 1% antibiotics. After the isolation passage, 5 × 10^5^ cells from each donor were grouped and pooled by donor age (<60 years, 60–75 years, and >75 years) and used for downstream applications. LSC from two, three, and seven donors, respectively, composed the LSC pools for age <60 years (mean 48 ± 7.07 years), 60–75 years (mean 64.33 ± 2.30 years), and >75 years (mean 83.42 ± 4.72 years) were used. Corneal epithelial cells were obtained by mechanical scrapping of the central corneal epithelium, avoiding the perilimbal region. Conjunctival epithelial cells were obtained by conjunctival epithelial biopsy and* in vitro* culture amplification of the explant in DMEM supplemented with 10% FCS and 1% antibiotics for 12 days.

### 2.3. Colony Forming Efficiency (CFE)

For CFE determination, 10 cells/cm^2^ of the pooled LSC were seeded in 35 mm diameter plates and cultured in SHEM for 14 days [34] with feeder-layer support. After that, cells were washed with 100 mM PBS with EDTA 2 mM for 30 sec to detach the feeder 3T3 cells. Colonies were fixed and stained with 0.5% crystal violet in methanol, scored according to previous criteria, and presented as a percentage after applying the previously described formula [35]. The diameter of each colony was measured using ImageJ software [36].

### 2.4. Quantitative Immunofluorescence (Q-IF)

This method is based on the Q-FIHC methodology for human LSC implemented before [37]. Pooled cells of each group (5 × 10^5^) were added to ThinPrep® PreservCyt solution (Hologic Iberia SL, Barcelona, Spain) for fixation and preservation. Cells were then transferred to slides using ThinPrep 3000 processor (Hologic), which allowed the cells to be seeded in a single plane without forming clumps. Slides were preserved in methanol until use, when three slides per condition were permeabilized, blocked, and then incubated with primary monoclonal antibody (mAbs) against p63 (clone BC4A4, Abcam, Cambridge, UK) for 60 min at 37°C in a humidified chamber. After several washes in 100 mM PBS solution, proper secondary antibody was added for 60 min at 37°C in a humidified chamber while phalloidin-TRITC (P1951, Sigma) was used to stain the cytoplasm to facilitate determination of cell diameters. The nuclei were counterstained with DAPI and slides were mounted with an antifading mounting medium (Vectashield, Vector Laboratories, CA, USA). Cells were observed in an epifluorescence microscope (BX61; Olympus R-FTL-T; Olympus America Inc., Center Valley, PA), coupled with a program for digital image acquisition (Olympus DP Controller Program). About 1000 cells were analyzed for p63 protein per condition. Slides incubated with the secondary antibody, in absence of primary specific mAbs, were used as negative controls for fluorescence settings. Also, conventional immunofluorescence for cytokeratin (CK) 12, CK3, and CK19 was carried out by processing the slides as described. The antibodies and concentrations used are detailed in Table 1.

### 2.5. Q-IF Image Processing

Images were processed with ImageJ [36] software, using a macro that automatically rested the background of every channel (blue for nuclei, red for cytoplasm, and green for p63+ cells) established before image acquisition with the negative controls. Next, the images were segmented (Laplacian of Gaussian plugin) and a binary mask was generated for every channel. Cells that touched each other were separated using watershed plugin after segmentation. The nuclei mask was then used to count the total number of cells. Only cells with blue and green staining in the corresponding channels were analyzed in the red channel for calculating the diameter. Cells were only counted if their cytoplasm was completely within the acquired field. The results gave the total number of cells, the number of p63+ cells with a diameter ≤10 *μ*m (small cells), and the number of p63+ cells with a diameter >10 *μ*m (big cells) [34].

### 2.6. Western Blot (WB) Analysis

Total cell extracts were dissolved in SDS-loading buffer, and the lysate (20 *μ*g of protein) was electrophoresed on a 12% SDS polyacrylamide gel (SDS-PAGE). The separated proteins were transferred for 90 min at 90 V to nitrocellulose transfer membranes (BD Biosciences, San José, CA) and then blocked for 1 h with 5% skimmed milk. Primary antibodies (Table 1) were incubated overnight at 4°C. After several washes, horseradish peroxidase-conjugated goat anti-mouse or swine anti-rabbit immunoglobulin (DakoCytomation, Denmark) was added for 90 min at room temperature. Protein bands were revealed using an enhanced chemiluminescence substrate (Biological Industries, Reactiva, Barcelona, Spain) and recorded on autoradiography film (Kodak Rochester, NY, USA). WB analysis was performed by digital scanning of the blots, followed by densitometric analysis with ImageJ [36]. Analysis of p63 was normalized to tubulin as the loading control.

### 2.7. mRNA Extraction and Quantitative Polymerase Chain Reaction (qPCR) Analysis

Total RNA was extracted from LSC, corneal epithelial cells, and conjunctival epithelial cells using RNA PureLink Mini Kit (Ambion, Invitrogen), following the manufacturer's instructions. The RNA concentration was measured using Tecan Infinite m200 Pro Absorbance Reader (Tecan, Männedorf, Switzerland). RNA (1 *μ*g) was reverse-transcribed using Superscript III (Invitrogen) according to the manufacturer's instructions. Then, cDNA (1 *μ*L) was used for qPCR in a final volume of 18 *μ*L with SYBR Green Reaction Mix (Invitrogen) and a 0.2 *μ*M primer concentration. The qPCR was performed using Step One (Applied Biosystems, Life technologies, Glasgow, UK) hardware and software. The expression level of target genes was normalized to internal 18s (rrn18s, TATAA Biocenter, Sweden) and represented as relative expression using 2^−ΔΔCt^ formula. The sequences and annealing temperatures of PCR primers are listed in Table 2.

### 2.8. Statistical Analysis

Experiments were performed in triplicate, at least. A two-tailed Student's *t*-test was run and *p* values <0.05 were considered statistically significant (PRISM, version 6.0 GraphPad Software, San Diego, CA). Results are presented as the mean ± standard error (MD ± SE) or, in the case of the qPCR analysis, mean ± standard deviation (MD ± SD).

## 3. Results

### 3.1. qPCR

**Figure 1:**
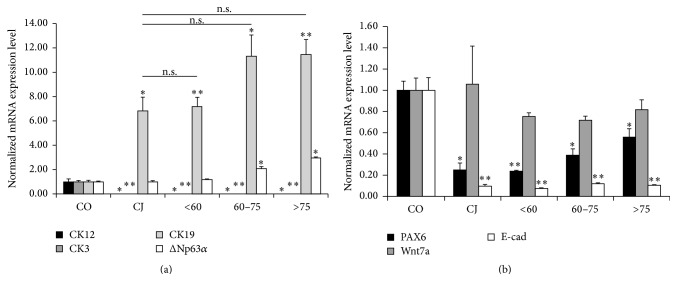
Comparative qPCR analysis of corneal progenitor and differentiation markers in LSC pools. (a) mRNA expression of CK12, CK3, CK19, and ΔNp63*α*. (b) mRNA expression of PAX6, Wnt7a, and E-cad. Results are presented as mean ± SE from 3 independent experiments. Statistical analysis was performed using two-tailed Student's *t*-tests (^*∗*^ *p* < 0.05; ^*∗∗*^ *p* < 0.01; and ns: not significant). CJ: conjunctiva cells; CK: cytokeratin; CO: corneal epithelial cells; E-cad: E-cadherin.

To characterize LSC we evaluated CK12, CK3, PAX6, Wnt7a, and E-cadherin (E-cad) mRNA levels as markers of corneal differentiation and CK19 and ΔNp63*α* mRNA levels as putative markers of stemness. LSC from donors aged 60–75 years and >75 years expressed significantly higher levels of ΔNp63*α* mRNA than cornea (CO) and conjunctiva (CJ), while LSC from donors <60 years showed no differences. In comparison with CO, LSC from all groups did not express CK12 and CK3 as well as CJ. CK19 mRNA levels were significantly higher in CJ and all LSC groups when compared to CO without significant differences between CJ and all LSC groups. E-cad and PAX6 mRNA levels were also significantly lower in LSC and CJ when compared to CO (Figure 1).

### 3.2. WB

**Figure 2:**
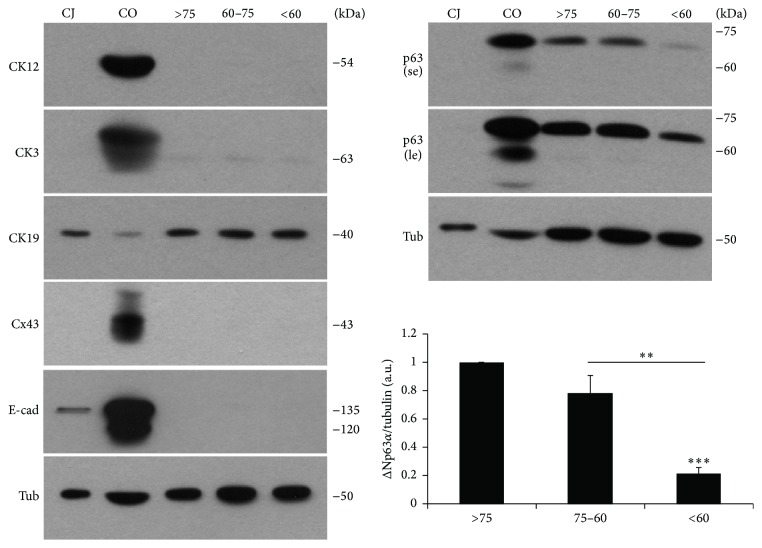
Comparative WB analysis of corneal progenitor markers and differentiation markers in LSC pools. No protein expression was detected for CK12, CK3, Cx43, or E-cad in each LSC pool, whereas expression was seen for CK19. Various isoforms of p63 were also present in the corneal cells (CO), but only ΔNp63*α* was seen in LSC, even at long exposure times. The graph shows the densitometric analysis of ΔNp63*α*. Results are presented as mean ± SE from 3 independent experiments. Statistical analysis performed using two-tailed Student's *t*-test (^*∗∗*^ *p* < 0.01; ^*∗∗∗*^ *p* < 0.001). CJ: conjunctiva cells; CK: cytokeratin; CO: corneal epithelial cells; Cx: connexin; E-cad: E-cadherin; le: long exposure; se: short exposure; tub: tubulin.

We also assayed the presence of CK12, CK3, E-cad, Cx43, CK19, and p63 by WB, which confirmed the qPCR results. CK12, CK3, and Cx43 protein were only present in CO and were absent in CJ and all LSC groups, confirming that these markers are highly specific for cornea. Interestingly, E-cad was observed in both its mature (120 KDa) and immature (135 KDa) [38] forms in CO, with only the immature form present in CJ, indicating that mature E-cad could be specific of corneal epithelial cells. CK19 was detected in CJ and all LSC groups, but with less protein detected in CO. The Δp63*α* isoform of p63 was detected in all LSC groups, but there was a significantly lower level of protein in younger donors (<60 years) when compared to the older donor groups. Various p63 isoforms (ΔNp63*α*, ΔNp63*β*, and ΔNp63*γ*) could be detected in CO but were not detected in CJ (Figure 2). With additional experiments (see Figure S1 of the Supplementary Material available online at http://dx.doi.org/10.1155/2016/3032128), comparing CO with different amounts of protein obtained from several CJ samples, it was noted that p63 was expressed at lower levels in CJ samples than in CO. In fact, p63 could not be detected when loading similar amounts of protein from CO and CJ cell cultures or even from CJ tissue, but it was detected when increasing amounts of CJ protein were loaded [39]. Also, various p63 isoforms could be detected in CJ samples, ΔNp63*α* being the most expressed.

### 3.3. Immunofluorescence

**Figure 3:**
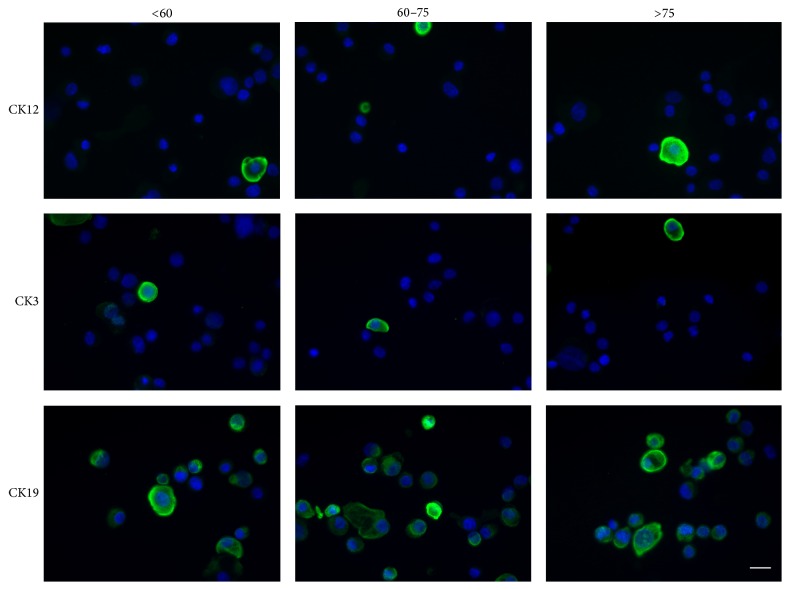
Immunofluorescence for CK12, CK3, and CK19 in LSC pools. Note the lack of expression of CK12 and CK3, with only sparse cells expressing these in certain fields. CK19 was expressed in each condition. Scale bar = 10 *μ*m.

Next, we performed indirect immunofluorescence for CK12, CK3, and CK19, which again corroborated the earlier results. LSC from each donor group were broadly negative for CK12 and CK3 but expressed CK19 (Figure 3), consistent with previous research [40, 41].

### 3.4. Q-IF

**Figure 4:**
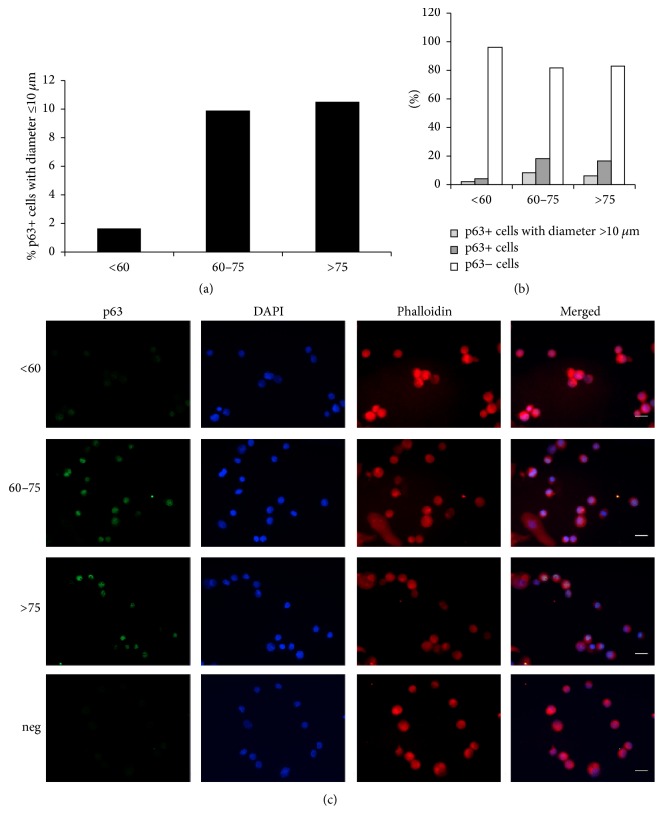
Quantitative immunofluorescence (q-IF) of p63 in LSC pools. (a) Histogram shows the percent of p63+ cells with diameters ≤10 *μ*m. LSC from aged donors showed >3% p63+ cells while younger donors had <3%. (b) Comparative histograms of p63+ cells with diameters >10 *μ*m, the overall p63+ cells (with diameters >10 *μ*m and ≤10 *μ*m), and the p63-cells. (c) Representative images of the analysis. Scale bar = 10 *μ*m. Results are presented as percent of total cells. *N*_<60_ = 973, *N*_60–75_ = 982, and *N*_>75_ = 1101.

LSC from donors aged 60–75 years and >75 years presented ≥3% of small-diameter p63+ cells, which is considered the minimum percentage that can assure favorable LSCT clinical outcomes [26]. Specifically, small p63+ cells were present in about 9.87% of LSC from donors aged 60–75 years and about 10.53% of donors aged >75 years. LSC from donors aged <60 years had far fewer small p63+ cells (1.64%) and therefore failed to meet the minimum acceptable criteria for LSC graft quality (Figure 4). These results showed a Pearson correlation of 0.97 when compared with the means of the Δp63*α* protein quantification in WB. With respect to the big p63+ cells, these accounted for 2.26% of LSC from donors aged <60 years, 8.24% of those from donors aged 60–75 years, and 6.17% of those from donors aged >75 years. Adding these results together, 3.9%, 18.12%, and 16.75% of LSC were p63+ in the donor groups aged <60 years, 60–75 years, and >75 years, respectively. These overall results agree with the quantitative data previously analyzed by Q-FIHC methodology [37].

### 3.5. CFE and Morphology of the Cell Cultures

**Figure 5:**
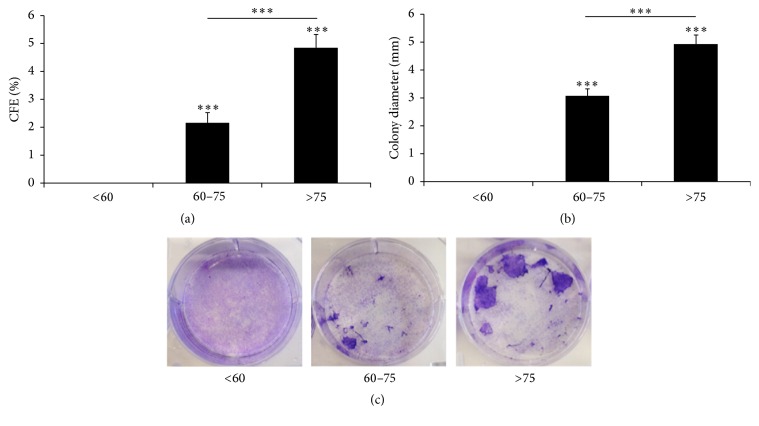
CFE with LSC pools. (a) Younger LSC donors did not show clones, and only 3T3 cells were seen as no washes were performed in this to prevent complete cell detachment. (b) Diameter of the colonies grown in (a): *N*_<60_ = 0, *N*_60–75_ = 13, and *N*_>75_ = 29. (c) Representative CFE images. The results are presented as mean ± SE of 6 independent experiments. Statistical analysis was performed using two-tailed Student's *t-*test (^*∗∗∗*^ *p* < 0.001).

**Figure 6:**
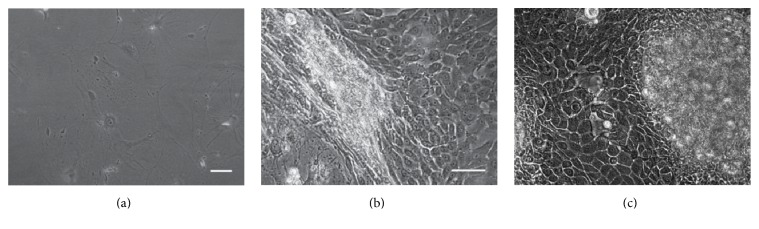
Micrographs of the clones from CFE. (a) No colonies grew in CFE of LSC form donors aged <60 years. (b) The morphology of the LSC clones from donors aged 60–75 years. (c) The morphology of the LSC clones from donors aged >75 years. Scale bar = 20 *μ*m.

**Figure 7:**
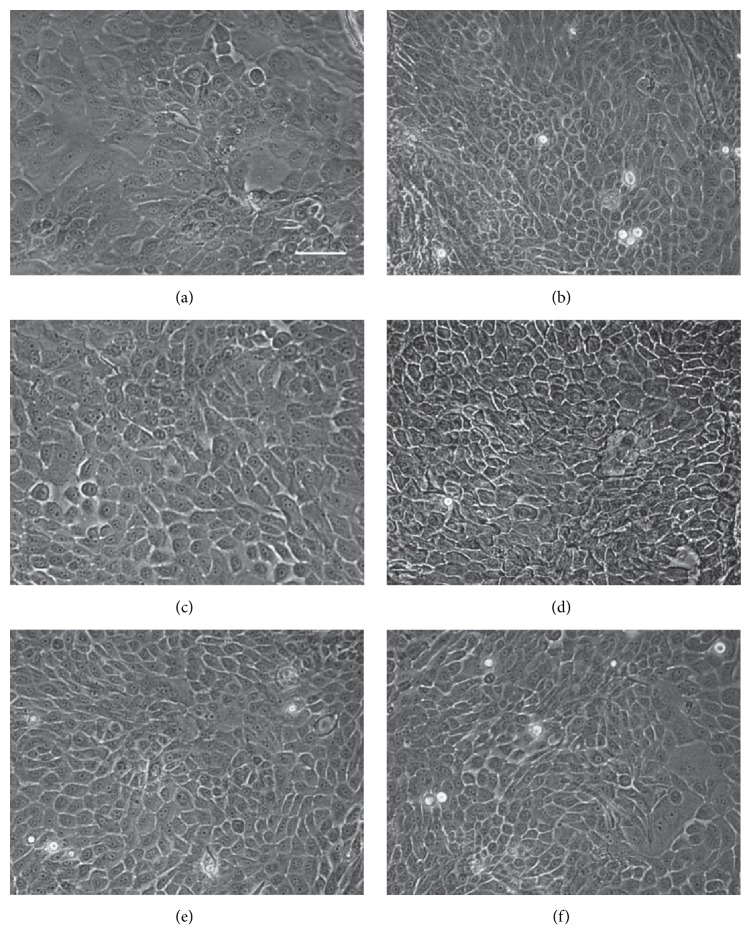
Representative micrographs of the morphology of LSC by donor age range at isolation passage. (a, b) Isolation passage of two donors aged <60 years. (c, d) Isolation passage of two donors aged 60–75 years. (e, f) Isolation passage of two donors aged >75 years. Scale bar = 20 *μ*m.

The CFE was higher for LSC from donors aged >75 years (4.83%) than from donors aged 60–75 years (2.16%). Similarly, the colony diameter presented higher values in the CFE assay of the donors aged >75 years. The CFE assay failed in the LSC from donors aged <60 years because no clones were detectable (Figures 5 and 6). Colonies from the group aged >75 years were classified as holoclones, while colonies from the group aged 60–75 years were classified as meroclones based on colony diameter [35]. The isolation passage of LSC cultures from each donor presented the characteristic morphology of LSC, showing small-sized polygonal cells with little cytoplasm [30] (Figure 7).

## 4. Discussion

It was previously observed that the niche of LSC changes with age, the area of the crypts of the palisades of Vogt being reduced after the age of 60 years. However, the levels of putative stem cell markers and telomere length or telomerase activity do not show differences between ages [42]. Our results fit with these findings, as LSC from donors older than 60 years showed higher levels of the putative stem cell marker p63. As assayed by WB, immunofluorescence, and qPCR, the LSC from each donor group were characterized, according to previous well-stablished statements [30], as they do not express corneal differentiation markers (CK12, CK3, Cx43, and E-cad) and express CK19, that despite being considered a conjunctival marker it is also expressed by LSC [28, 29, 43, 44]. Nevertheless, LSC from donors younger than 60 years expressed p63 cells at a percentage <3% as tested by Q-IF, consistent with the results of qPCR and WB that also showed lower levels of this putative stem cell marker. LSC from older donors (both 60–75 years and >75 years groups) presented a percentage of p63 >3%, showing again the consistency between the Q-IF, qPCR, and WB results. Moreover, the quantitation of p63 obtained by Q-IF was comparable to that previously found with Q-FIHC [37]. Our results also point out the validity of WB for the identification of LSC as commented before [45]. Our data supports the notion that isoform Δp63*α* is very specific for LSC, while isoforms Δp63*β* and Δp63*γ* are lacking in LSC [46]. These 3 isoforms are all present in cornea, the Δp63*α* isoform being the most common [47]. Previous reports have demonstrated p63 protein expression in conjunctival tissue [47, 48], and we have also noticed that its expression in conjunctival cell cultures is lower than in corneal epithelial cells [39].

As commented, it was observed that LSC cultures containing ≥3% p63+ cells can lead to successful LSCT with a rate of 74.6%, whereas cultures with <3% p63+ cells have a success rate of only 8% [26]. Although LSCT failures can also be caused by improper selection of patients with high levels of ocular damage [49], the percentage of p63+ cells seems to be, at present, a key factor predicting clinical outcomes in LSCT [50]. The fact that the younger group (<60 years) presented a lower percentage of p63+ cells and an absence of CFE may be explained by interdonor variation and the low number of young donors, a limitation in this study. However, we showed that LSC cultures from older donors (≥60 years) did meet the quality criterion for graftable limbal cultures in terms of percentage of p63+ cells predicting possible clinical outcomes if used in LSCT. Our results are consistent with previous findings suggesting that LSC could be used from older cadaveric donors without affecting the clinical success of the procedure as donor age did not correlate with limbal explant outgrowth [51].

Although CFE is not a sufficient parameter that could predict culture potency [26], we showed that both CFE and colony diameter correlated somewhat with well-established stemness parameters, such as p63. In our study, CFE assay from the LSC of donors aged <60 years failed, demonstrating that CFE can give complementary information about LSC culture quality. We also showed that cultures with good morphology in the isolation passage did not necessarily correlate with graft quality criteria.

Finally, it was also demonstrated that, in clinical situations where it is possible to choose between explant or cell suspension for LSC isolation, the cell suspension method was the best option for LSC enrichment [52–54]. A common disadvantage of the explant over the cell suspension method is the small amount of cells obtained because of the low proliferative rate in the former [53–55]. Moreover, LSC isolated by cell suspension can help reduce contamination by other cell types, such as fibroblasts [52]. As there were no differences in clinical outcomes between LSC autotransplantation and allotransplantation [7, 8, 11], we suggest that using cell pools from suitable donors isolated by cell suspension could mitigate interdonor variation and increase the amount of cells. This would allow together the allotransplantation and the graft quality criteria screenings (as p63 and CK3 quantification) before implantation, which might improve clinical outcomes.

## 5. Conclusion

In summary, we demonstrate that donor age is not enough criterion for predicting the behavior of the culture, showing that LSC from aged donors can be a potential source of LSC for allogenic transplantation based on the expression of putative stemness markers and CFE potential. Moreover, our research highlights the need to evaluate each donor in terms of LSC culture quality.

## Supplementary Material

Decreased expression of p63 protein in conjunctiva compared to cornea.

## Figures and Tables

**Table 1:** Primary and secondary antibodies.

Antibody	Source	Isotype	Clone	Application	Dilution
Connexin 43	Santa Cruz Lab	Rabbit IgG	H-150	WB	1/1000
Cytokeratin 12	Abcam	Rabbit IgG	EPR1609(2)	WB	1/2000
Cytokeratin 12	Santa Cruz Lab	Goat IgG	L-20	IFI	1/100
Cytokeratin 19	Boehringer Mannheim	Mouse IgG1	170.2.14	WB, IFI	1/1000, 1/50
Cytokeratin 3	Millipore	Mouse IgG1	AE5	WB, IFI	1/2500, 1/100
E-cadherin	BD Transduction Laboratories	Mouse IgG2a	36	WB	1/2000
p63	Millipore	Mouse IgG2a	4A4	WB	1/1250
p63	Abcam	Mouse IgG2a	BC4A4	IFI	1/50
Tubulin	Sigma	Mouse IgG1	DM1A	WB	1/2000
Phalloidin-TRITC	Sigma			IFI	1/2000
Goat anti-rabbit	Sigma	Goat		WB	1/2500
Rabbit anti-mouse	Sigma	Rabbit		WB	1/2500
Goat A488	Invitrogen Life Technologies	Goat		IFI	1/1000
Mouse A488	Invitrogen Life Technologies	Mouse		IFI	1/1000

WB: Western blot; IFI: indirect immunofluorescence.

**Table 2:** Primers and sequences.

Target	Gene	Accession number		Primer sequence (5′-3′)	Annealing temperature
Cytokeratin 12	CK12	NM_000223	F	TGGTCATGTTGGTCTTTGTAAC	55°C
			R	ACTTCTCTCTATGCTCTTGACA	
Cytokeratin 3	CK3	NM_057088	F	GAGCGGGAACAGATCAAGAC	55°C
			R	GGTAGCTCCGCAGGTAGTTG	
Cytokeratin 19	CK19	NM_002276	F	TGAGTGACATGCGAAGCCAAT	55°C
			R	ACCTCCCGGTTCAATTCTTCA	
Δp63*α*	TP63	NM_003722.4	F	GAAACGTACAGGCAACAGCA	60°C
			R	GCTGCTGAGGGTTGATAAGC	
E-cadherin	E-cad	NM_004360	F	GCCTCCTGAAAAGAGAGTGGAAG	60°C
			R	TGGCAGTGTCTCTCCAAATCCG	
Wnt7a	WNT7A	NM_004625.3	F	CATAGGAGAAGGCTCACAAATGG	55°C
			R	CGGCAATGATGGCGTAGGT	
PAX6	PAX6	NM_000280.4	F	ATAACCTGCCTATGCAACCC	55°C
			R	GGAACTTGAACTGGAACTGAC	
RNA-18S	18S	NR_003286.2	F	TATA center	60°C
			R	TATA center	

F: forward; R: reverse.
