# Supplementary material for: Limbal Stem Cells from Aged Donors Are a Suitable Source for Clinical Application
Source: Stem Cells Int. 2016 Nov 30;2016:3032128. doi: 10.1155/2016/3032128 (PMC5155095; doi:10.1155/2016/3032128)

SUPPLEMENTARY DATA

Figure S1. Levels of p63 protein expression in cornea and conjunctiva by WB. Different amounts of protein from conjunctiva explant cell cultures (lanes 2, 3 and 5) and from conjunctival tissue biopsies (lanes 4 y 6) were loaded and compared with extracts of corneal cells (lane 1). Tub, tubulin.

Figure S1

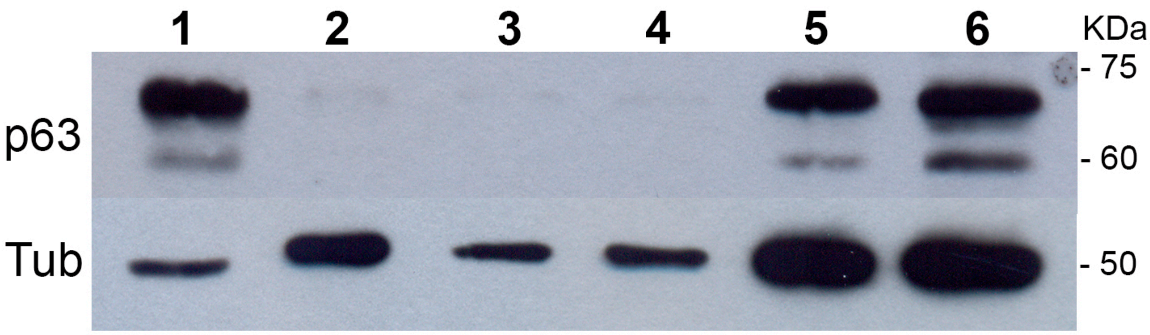

Supplement: Supplementary file 1 — Decreased expression of p63 protein in conjunctiva compared to cornea. [file 3032128.f1.pdf]
